# Impact of the COVID-19 pandemic on lung-protective ventilation practice in critically ill patients with respiratory failure: a retrospective cohort study from a New England healthcare network

**DOI:** 10.1186/s13054-024-04982-4

**Published:** 2024-07-04

**Authors:** Ricardo Munoz-Acuna, Elena Ahrens, Aiman Suleiman, Luca J. Wachtendorf, Basit A. Azizi, Simone Redaelli, Tim M. Tartler, Guanqing Chen, Elias N. Baedorf-Kassis, Maximilian S. Schaefer, Shahla Siddiqui

**Affiliations:** 1 Center for Anesthesia Research Excellence (CARE), Beth Israel Deaconess Medical Center, Harvard Medical School, Boston, MA USA; 2 Department of Anesthesia, Critical Care and Pain Medicine, Beth Israel Deaconess Medical Center, Harvard Medical School, 330 Brookline Avenue, Boston, MA 02215 USA; 3 Department of Anesthesia, Intensive Care and Pain Management, Faculty of Medicine, University of Jordan, Amman, Jordan; 4 School of Medicine and Surgery, University of Milano-Bicocca, Milan, Italy; 5 Department of Pulmonary, Critical Care and Sleep Medicine, Beth Israel Deaconess Medical Center, Harvard Medical School, Boston, MA USA; 6 Department of Anesthesiology, Duesseldorf University Hospital, Duesseldorf, Germany

## Correspondence (785/800)

To the Editor—Before the Coronavirus Disease 2019 (COVID-19) pandemic, over three million patients in the United States of America (USA) suffered from hypoxemic respiratory failure annually. COVID-19-related hypoxemic respiratory failure required admission to the intensive care unit (ICU) in nearly 30% of cases and mechanical ventilation for more than 10% of patients, leading to strain in the healthcare system [1]. Previous evidence suggested an increased mortality in non-COVID-19 patients related to increased health-care strain. The question remains whether patient care, and especially best-practice mechanical ventilation management, was also affected by the pandemic [2]. We hypothesized that the COVID-19 pandemic with its consequences on healthcare strain and staffing shortages affected ventilator management and lung-protective ventilation (LPV) practice patterns in patients with hypoxemic respiratory failure.

Mechanically ventilated patients admitted to the ICUs of Beth Israel Deaconess Medical Center, Boston, MA, USA, with hypoxemic respiratory failure between January 2018 and December 2021 were included. Hypoxemic respiratory failure was defined as a ratio of partial arterial pressure of oxygen to fraction of inspired oxygen (P/F) ≤ 300 at the first available blood gas analysis. Patients with a duration of mechanical ventilation < 12 h or with missing data on confounding variables were excluded. LPV was defined as the simultaneous presence of a plateau pressure (P_plat_) of < 30 cmH_2_O, a driving pressure ≤ 15 cmH_2_O, as well as tidal volumes (Vt) of 4–8 ml per kilogram of predicted body weight (PBW) [3]. Parameter recordings within the first two hours of mechanical ventilation were excluded to avoid artefacts from the initial patient transfer and stabilisation period. We examined changes in LPV practices during and pre-pandemic periods using an interrupted time series analysis with quarterly time points. The second quarter of the year 2020 (April to June) was established as ‘start of intervention period’ since April 2020 was the month when COVID-19 patients reached the proportional majority in ICU occupation in line with the pandemic transmission consolidation in the USA [4]. Analyses were adjusted for patient baseline characteristics (age, sex, respiratory system compliance, P/F ratio, and Elixhauser Comorbidity Index).

**Fig. 1:**
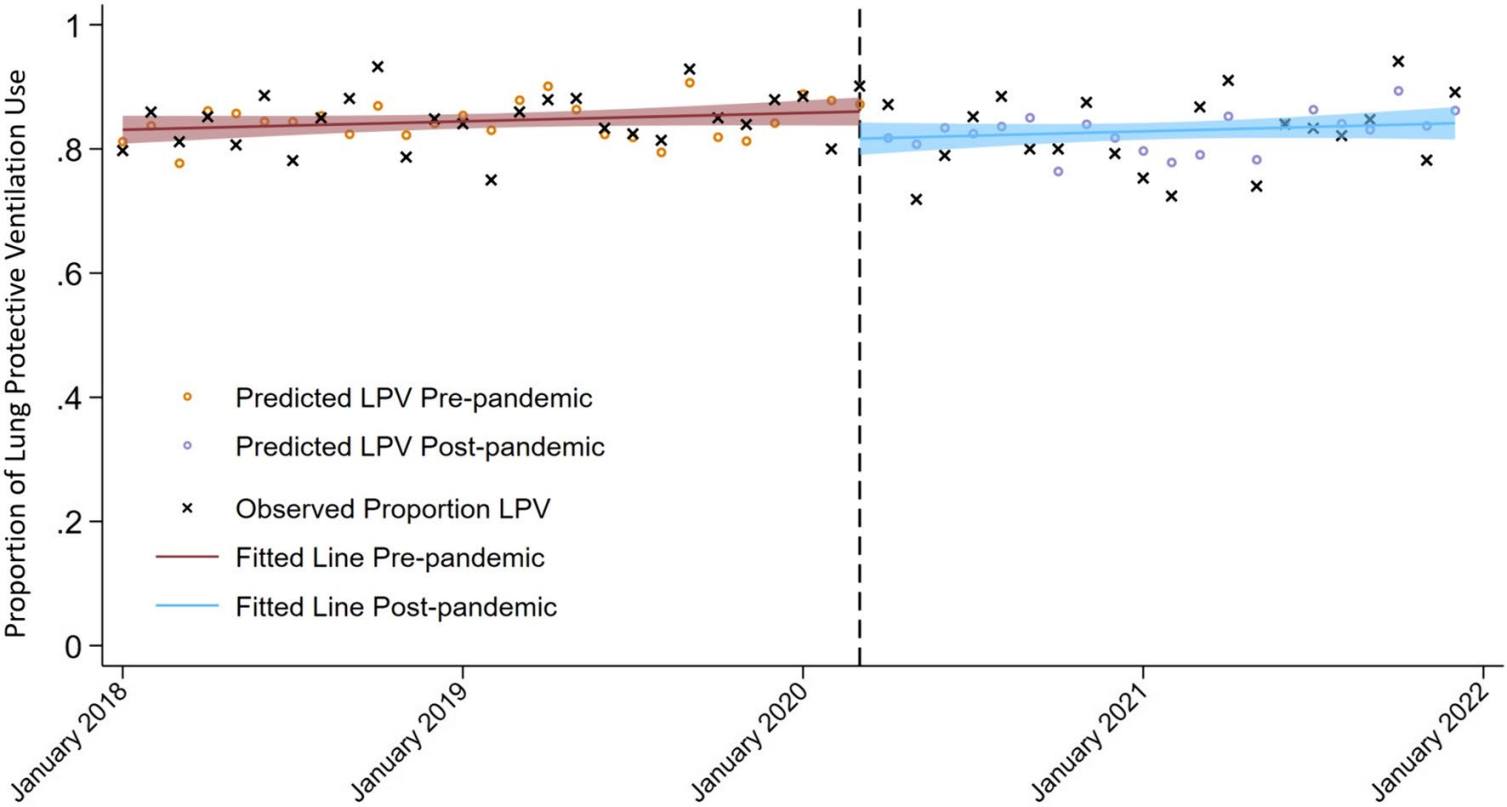
Interrupted Time Series Analysis. The multivariate linear prediction is depicted in bold lines with its respective 95% confidence interval and the adjusted prediction is presented as hollow circles, black crosses represent the observed LPV. The pre-pandemic period is represented in red, and the pandemic period is shown in blue. Abbreviations: LPV: Lung-protective ventilation

Among 2965 included patient cases, 1381 (46.6%) were admitted pre-pandemic and 1,584 (54.4%) during the pandemic. Overall, after onset of the pandemic, between 3.3% and 77.9% of patients per month were COVID-19 positive with an overall of 386 (28%) patients included. Detailed patient characteristics, ventilator parameters and demographics are included in the Supplemental Document 1, Tables S1, S2. Prior to the pandemic, there was an increasing trend in the utilization of LPV (absolute increase of 0.8% per quarter; 95% CI 0.3–1.4%; *p* = 0.006, Fig. 1). During the first three months after the pandemic onset, there was an absolute decrease of − 3.2% (95% CI − 6.3 to − 0.2%; *p* = 0.049) in the utilization of LPV in comparison to the preceding quarter before the pandemic (January–March 2020). Subsequently, the utilization of LPV did not change over the course of the broader COVID-19 pandemic period (April–December 2021, absolute decrease − 0.1% per quarter after the onset of the pandemic; 95% CI − 0.7 to 0.5; *p* = 0.62).

These findings of a discrete ascent in LPV practices in the ICU before the onset of the COVID-19 pandemic align with other studies reporting a wide application of mechanical ventilation using low Vt and driving pressures [5]. The decrease in the utilization of LPV after the onset of the COVID-19 pandemic potentially reflects a systemic disruption of resource allocation after March 2020, including protective equipment supplies, ventilators, and hospital staff. Medical centers across the USA suffered from staffing shortages that might have contributed to worsened patient outcomes and suboptimal respiratory care. Furthermore, it might be attributed to a higher prevalence of patients with severe lung disease in the ICU as reflected by the lower P/F ratio in the pandemic period (Tables S1, S2). Ventilation management adherent to LPV protocols can be difficult in patients with worsening respiratory system compliance, and severe hypercapnia or hypoxemia.

The generalizability to other settings is limited by the use of data from one academic hospital network in New England. Our findings now provide a rationale to investigate the impact of ICU stress on quality of care in different scenarios as well as hospital settings and geographical locations.

In conclusion, the COVID-19 pandemic may have influenced the existing trend in the implementation of LPV strategies in critically ill patients. The data suggest that the overall trend in the utilisation of LPV remained stable throughout the pandemic, which could indicate some resilience and adaptability in ICU practices. However, the findings also imply that patients with hypoxemic respiratory failure were less likely to receive LPV, though these observations should be interpreted with caution given the study's retrospective design. Further research is needed to confirm these trends.

### Supplementary Information

Supplementary file 1

## Data Availability

The datasets generated and/or analyzed during the current study are not publicly available due data compliance and privacy policies but are available from the corresponding author on reasonable request by a qualified researcher.

## Abbreviations

COVID-19: Coronavirus Disease 2019
ICU: Intensive care unit
LPV: Lung-protective ventilation
PBW: Predicted body weight
P/F ratio: Ratio of the partial pressure of arterial oxygen to the fraction of inspired oxygen
P_plat_: Plateau pressure
Vt: Tidal volume
