# Supplementary material for: Impact of the COVID-19 pandemic on lung-protective ventilation practice in critically ill patients with respiratory failure: a retrospective cohort study from a New England healthcare network
Source: Crit Care. 2024 Jul 4;28:219. doi: 10.1186/s13054-024-04982-4 (PMC11225354; doi:10.1186/s13054-024-04982-4)
Supplement: Supplementary file 1 [file 13054_2024_4982_MOESM1_ESM.docx]

**Supplemental Document 1**

**Impact of the COVID-19 pandemic on lung-protective ventilation practice in critically ill patients with respiratory failure: A retrospective cohort study from a New England healthcare network**

*Ricardo Munoz-Acuna, MD*^1,2^*, Elena Ahrens, cand.med.*^1,2^*, Aiman Suleiman, MD, MSc*^1,2,3^*, Luca J. Wachtendorf, MD^1,2^, Basit A. Azizi, cand.med.*^1,2^*,* *Simone Redaelli, MD^1,2,4^, Tim M. Tartler, MD*^1,2^*, Guanqing Chen, PhD*^1,2^*, Elias N. Baedorf-Kassis, MD*^1,5^*,* *Maximilian S. Schaefer, MD, PhD*^1,2,6^*, Shahla Siddiqui, MD, MBBS, D ABA, MSc, FCCM*^2^

^1^Center for Anesthesia Research Excellence (CARE), Beth Israel Deaconess Medical Center, Harvard Medical School, Boston, MA, USA.

^2^Department of Anesthesia, Critical Care and Pain Medicine, Beth Israel Deaconess Medical Center, Harvard Medical School, Boston, MA, USA.

^3^Department of Anesthesia, Intensive Care and Pain Management, Faculty of Medicine, University of Jordan, Amman, Jordan.

^4^School of Medicine and Surgery, University of Milano-Bicocca, Milan, Italy.

^5^Department of Pulmonary, Critical Care and Sleep Medicine, Beth Israel Deaconess Medical Center, Harvard Medical School, Boston, MA, USA.

^6^Department of Anesthesiology, Duesseldorf University Hospital, Duesseldorf, Germany.

*To respect space limitations in the main manuscript, we present detailed description of methods and analyses including the secondary and sensitivity analyses.*

**TABLE OF CONTENTS**

[**SECTION S1: SUPPLEMENTAL METHODS** 3](#_Toc168050926)

[**S1.1 Data sources** 3](#_Toc168050927)

[**S1.2 Exposure and outcome definitions** 3](#_Toc168050928)

[**S1.3 Confounder model** 4](#_Toc168050929)

[**S1.4 Interrupted time series model** 4](#_Toc168050930)

[**S1.5 Additional timeframes of mechanical ventilation** 5](#_Toc168050931)

[**S1.6 Congestive heart failure as independent predictor** 6](#_Toc168050932)

[**SUPPLEMENTARY TABLES** 8](#_Toc168050933)

[**Table S1. Patient characteristics and distribution of variables** 8](#_Toc168050934)

[**Table S2. Patient characteristics and distribution of variables of the last pre-pandemic quarter and the first COVID-19 pandemic quarter** 10](#_Toc168050935)

[**SUPPLEMENTARY FIGURES** 13](#_Toc168050936)

[**Figure S1. Study flow diagram** 13](#_Toc168050937)

[**Figure S2. Pattern of Lung-Protective Ventilation after Pandemic end** 14](#_Toc168050938)

[**Figure S3. Interrupted Times Series Analyses Model** 15](#_Toc168050939)

**REFERENCES**……………………**…………………………………………………………….13**

# **SECTION S1: SUPPLEMENTAL METHODS**

## **S1.1 Data sources**

Data were collected from routine clinical care at Beth Israel Deaconess Medical Center, Boston, Massachusetts, United States of America. Ventilator parameter data during spontaneous breathing were not included. Laboratory data and mechanical ventilation parameters were obtained from the Metavision intensive care unit (ICU) system and Respiratory Therapy database (1). These data were complemented with the billing registry Casemix, as well as information from the Admission Discharge Transfer (ADT) database. Further, data were merged with International Classification of Diseases (9^th^/10^th^ Revision, Clinical Modification [ICD-9/10-CM]) diagnostic codes billed within the healthcare network from Casemix (2). Data pertaining to admission and discharge information, as well as demographics, were obtained from both Casemix and ADT. All patient data were merged into a single combined dataset after strict de-identification.

## **S1.2 Exposure and outcome definitions**

The exposure variable was an ICU admission during the second quarter of the year 2020 or later. The exposure in the secondary analysis was defined as a Coronavirus disease 2019 (COVID-19) diagnosis, as identified by the ICD-9/10-CM code U07.1, or a positive polymerase chain reaction (PCR) laboratory for the severe acute respiratory syndrome coronavirus 2 (SARS-CoV-2). The outcome was lung-protective ventilation (LPV), defined as a simultaneous administration of a mean tidal volume of 4–8 ml per kilogram of predicted body weight (PBW), a mean driving pressure (calculated as plateau pressure [P_plat_] minus the total positive end-expiratory pressure) ≤ 15 cmH_2_O and a mean P_plat_ less than 30 cmH2O (calculated utilising a standardised end-inspiratory hold manoeuvres as part of clinical routine) during the first 22 hours of invasive mechanical ventilation excluding the first two hours after ICU admission to exclude parameters utilized during the initial transfer and care of the patient.

## **S1.3 Confounder model**

The applied analyses were adjusted for *a priori* defined confounding variables. Categorization into quintiles or clinically relevant groups was performed for variables demonstrating a nonlinear association with the outcome. Confounding variables included quintiles of age, standardized static respiratory system compliance, the lowest documented ratio of partial pressure of oxygen in arterial blood (PaO_2_) to a fraction of inspired oxygen (FiO_2_), and the Elixhauser Comorbidity Index (3,4). Sex was included as a binary variable.

## **S1.4 Interrupted time series model**

The interrupted time series analyses were fitted using the yearly quarters as individual observation points. Further, the aforementioned confounder model was accounted for in each time point using the mean of the observed values. The model was fitted using the Stata package “*ACTEST*” to perform Cumby-Huizinga (Breusch-Godfrey) general test for autocorrelation and to test for autocorrelation across different lags in our model (5). In our model, we found a significant autocorrelation at lag of 1 (p=0.003), where the error term followed a first-order autoregressive process. By including this first-order lag, we aim to control for the influence that the outcome variable's immediate past value might exert on its current value. This is essential in our analysis to ensure that the regression coefficients associated with other variables of interest, such as the intervention effect, are not confounded by this temporal dependency. The estimates produced were obtained using the Stata package “ITSA” and are further described in *Figure S3* (6). The post intervention trend is calculated with the linear combination of β1+ β3 (7).

## **S1.5 Additional timeframes of mechanical ventilation**

In order to verify the robustness of our primary findings we investigated the effect of the COVID-19 pandemic on the use of LPV including data within an extended time frame for 48 (i) and 72 hours (ii) yielding the following results, respectively:

(i) When the analysis period was contemplating up to 48 hours of mechanical ventilation we observed prior to the pandemic, there was an increasing trend in the utilisation of LPV (absolute increase of 0.8% per quarter; 95% CI 0.2–1.3%; p=0.01). During the first three months after the pandemic onset, there was an absolute decrease of -3.2% (95% CI -6.3 to -0.1%; p=0.04) in the utilisation of LPV in comparison to the preceding quarter before the pandemic (January to March 2020). Subsequently, the utilisation of LPV did not change over the course of the broader COVID-19 pandemic period (April to December 2021, absolute decrease -0.01% per quarter after the onset of the pandemic; 95% CI -0.6 to 0.6; p=0.963)

(ii) When the analysis period included up to 72 hours of mechanical ventilation the results of our analyses showed that prior to the pandemic, there was an increasing trend in the utilisation of LPV (absolute increase of 1.0% per quarter; 95% CI 0.8–1.2%; p<0.001). During the first three months after the pandemic onset, there was an absolute decrease of -2.2% (95% CI -6.0 to -0.8%; p=0.01) in the utilisation of LPV in comparison to the preceding quarter before the pandemic (January to March 2020). Subsequently, the utilisation of LPV did not change over the course of the broader COVID-19 pandemic period (April to December 2021, absolute decrease -0.1% per quarter after the onset of the pandemic; 95% CI -0.3 to 0.1; p=0.506)

## **S1.6 Congestive heart failure as independent predictor**

To further confirm our results and avoid biassed estimates due to the difference in proportion of patients diagnosed with congestive heart failure (650 [41%] patients in the pre-pandemic period compared versus 475 [34.4%] in the pandemic period ) we conducted the analysis in a model additionally adjusting congestive heart failure as an independent variable as well as in a cohort excluding patients with a congestive heart failure diagnosis which resulted in the following:

(i) In a model additionally confounding for congestive heart failure, prior to the pandemic, there was an increasing trend in the utilisation of LPV (absolute increase of 0.9% per quarter; 95% CI 0.6–1.2%; p<0.001). During the first three months after the pandemic onset, there was an absolute decrease of -3.2% (95% CI -6.0 to -0.5%; p=0.03) in the utilisation of LPV in comparison to the preceding quarter before the pandemic (January to March 2020). Subsequently, the utilisation of LPV did not change over the course of the broader COVID-19 pandemic period (April to December 2021, absolute decrease -1.9% per quarter after the onset of the pandemic; 95% CI -0.4 to 0.0003; p=0.08).

ii) In the cohort excluding patients with a diagnosis of congestive heart failure we observed that prior to the pandemic, there was an increasing trend in the utilisation of LPV (absolute increase of 2.6% per quarter; 95% CI 1.1–4.2%; p=0.005). During the first three months after the pandemic onset, there was an absolute decrease of -6.9% (95% CI -13.7 to -0.0004%; p=0.049) in the utilisation of LPV in comparison to the preceding quarter before the pandemic (January to March 2020). Subsequently, the utilisation of LPV changed over the course of the broader COVID-19 pandemic period (April to December 2021) by -1.4% per quarter.

# **SUPPLEMENTARY TABLES**

## **Table S1. Patient characteristics and distribution of variables**

|  | **Pre-pandemic period**  **n = 1 584** | **COVID-19 pandemic**  **n = 1 381** | **Standardized**  **difference** |
| --- | --- | --- | --- |
| **Age, years** | 66 (56 – 75) | 64 (55 – 73) | -0.11 |
| **Sex, female** | 601 (37.9%) | 485 (35.1%) | 0.07 |
| **BMI, kg/m^2^** | 29.1 (24.6 –34.5) | 28.5 (24.5 –33.8) | 0.03 |
| **Elixhauser comorbidity score** | 18 (10 – 26) | 19 (11 – 27) | 0.08 |
| **Congestive heart failure** | 650 (41.0%) | 475 (34.4%) | -0.14 |
| **Diabetes mellitus, without end organ damage** | 165 (10.4%) | 117 (8.5%) | -0.07 |
| **Chronic pulmonary disease** | 408 (25.8%) | 336 (24.3%) | -0.03 |
| **Renal failure** | 440 (27.8%) | 376 (27.2%) | -0.01 |
| **Hypertension** | 502 (31.7%) | 437 (31.6%) | -0.00 |
| **Pulmonary circulation disorders** | 277 (17.5%) | 235 (17.0%) | -0.01 |
| **P/F ratio** | 156 (105 – 212) | 130.0 (91 – 192) | -0.04 |
| **LPV** | 1 339 (84.5%) | 1 149 (83.2%) | -0.04 |
| **DP ≤15, cmH_2_O** | 1 448 (91.4%) | 1 231 (89.1%) | -0.08 |
| **VT 4-8 ml/kg PBW** | 1 488 (94.0%) | 1 329 (96.2%) | 0.11 |
| **Plateau pressure <30*** | 1 537 (97%) | 1 312 (95%) | -0.10 |
| **Vt, ml/kg PBW** | 6 (6 – 7) | 6 (6 – 7) | 0.02 |
| **DP, cmH_2_O** | 11 (9 – 13) | 11 (10 – 13) | 0.10 |
| **PEEP, cmH_2_O** | 7 (5 – 10) | 9 (6 – 12) | 0.33 |
| **PIP, cmH_2_O** | 21 (18 – 25) | 24 (20 – 28) | 0.37 |
| **Plateau pressure, cmH_2_O** | 19 (16 – 22) | 21 (17 – 24) | 0.31 |
| **Normalized respiratory system static compliance,**  **ml/ (cmH_2_O kg)** | 0.6 (0.5 – 0.7) | 0.6 (0.5 – 0.7) | 0.01 |

*Data are presented as number (prevalence in %) or median (IQR).*

*Abbreviations: BMI: Body mass index; DP: Driving pressure; LPV: Lung-protective ventilation; PEEP: Positive end-expiratory pressure; P/F ratio: Ratio of the partial pressure of arterial oxygen to the fraction of inspired oxygen; PIP: Peak inspiratory pressure; PBW: Predicted body weight; Vt: Tidal volume; COVID-19: Coronavirus Disease 2019.*

**Table S2. Patient characteristics and distribution of variables of the last pre-pandemic quarter and the first COVID-19 pandemic quarter**

|  | **Last pre-pandemic quarter**  **n = 183** | **First COVID-19 pandemic quarter**  **n = 242** | **Standardized**  **difference** |
| --- | --- | --- | --- |
| **Age, years** | 64 (52 - 75) | 64 (56 - 73) | 0.069 |
| **Sex, female** | 68 (37.2%) | 94 (38.8%) | -0.035 |
| **BMI, kg/m^2^** | 28.9 (24.5 - 33.9) | 30.0 (25.7 - 35.7) | 0.117 |
| **Elixhauser comorbidity score** | 17 (9 - 25) | 17 (11 - 24) | -0.052 |
| **Congestive heart failure** | 66 (36.1%) | 64 (26.4%) | -0.208 |
| **Diabetes mellitus, without end organ damage** | 21 (11.5%) | 20 (8.3%) | -0.108 |
| **Chronic pulmonary disease** | 44 (24.0%) | 51 (21.1%) | -0.071 |
| **Renal failure** | 42 (23.0%) | 55 (22.7%) | -0.005 |
| **Hypertension** | 69 (37.7%) | 84 (34.7%) | -0.062 |
| **Pulmonary circulation disorders** | 30 (16.4%) | 31 (12.8%) | -0.101 |
| **P/F ratio** | 158 (100 - 213) | 122 (88 - 178) | -0.342 |
| **LPV** | 159 (86.9%) | 198 (81.8%) | -0.139 |
| **DP ≤15, cmH_2_O** | 166 (90.7%) | 215 (88.8%) | -0.062 |
| **VT 4-8 ml/kg PBW** | 178 (97.3%) | 236 (97.5%) | 0.016 |
| **Plateau pressure <30*** | 178 (97.3%) | 219 (90.5%) | -0.285 |
| **Vt, ml/kg PBW** | 6 (6 - 7) | 6 (6 - 7) | -0.118 |
| **Driving pressure, cmH_2_O** | 11 (9 - 13) | 11.5 (10 - 13) | 0.185 |
| **PEEP, cmH_2_O** | 8 (5 - 11) | 11 (8 - 14) | 0.606 |
| **PIP, cmH_2_O** | 22 (18 - 27) | 26 (23 - 31) | 0.634 |
| **Plateau pressure, cmH_2_O** | 20 (17 - 23) | 23 (20 - 27) | 0.560 |
| **Normalized respiratory system static compliance,**  **ml/ (cmH_2_O kg)** | 0.6 (0.5 - 0.7) | 0.6 (0.5 - 0.7) | -0.181 |

*Data are presented as number (prevalence in %) or median (IQR).*

*Abbreviations: BMI: Body mass index; DP: Driving pressure; LPV: Lung-protective ventilation; PEEP: Positive end-expiratory pressure; P/F ratio: Ratio of the partial pressure of arterial oxygen to the fraction of inspired oxygen; PIP: Peak inspiratory pressure; PBW: Predicted body weight; Vt: Tidal volume; COVID-19: Coronavirus Disease 2019.*

# **SUPPLEMENTARY FIGURES**

## **Figure S1. Study flow diagram**


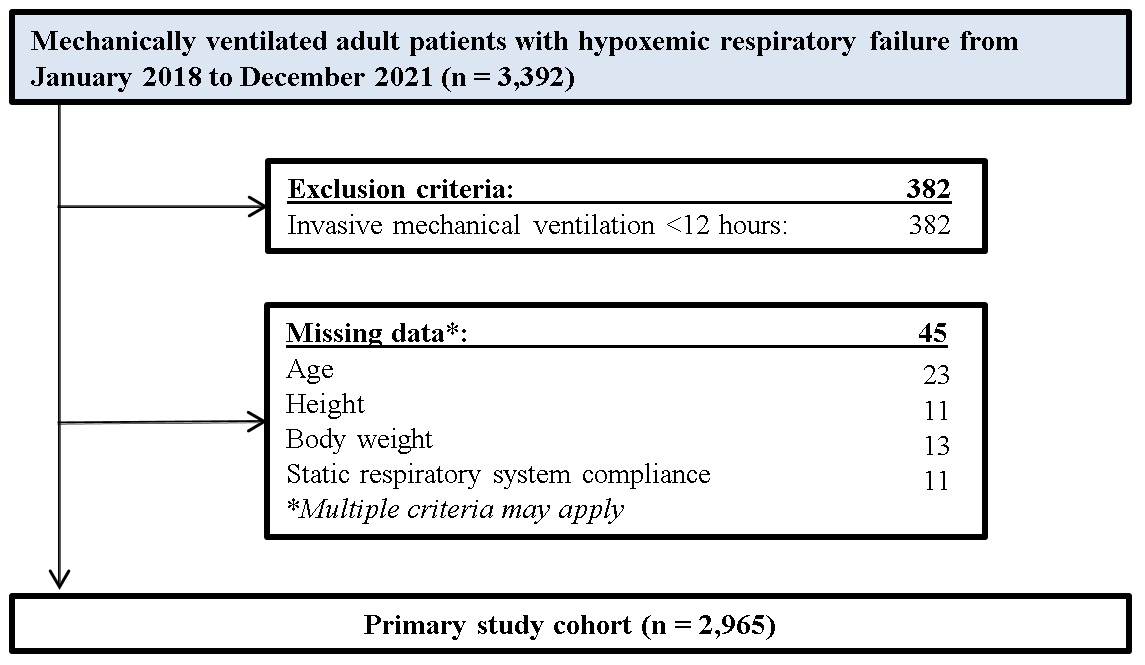


## **Figure S2. Pattern of Lung-Protective Ventilation after Pandemic end**

**
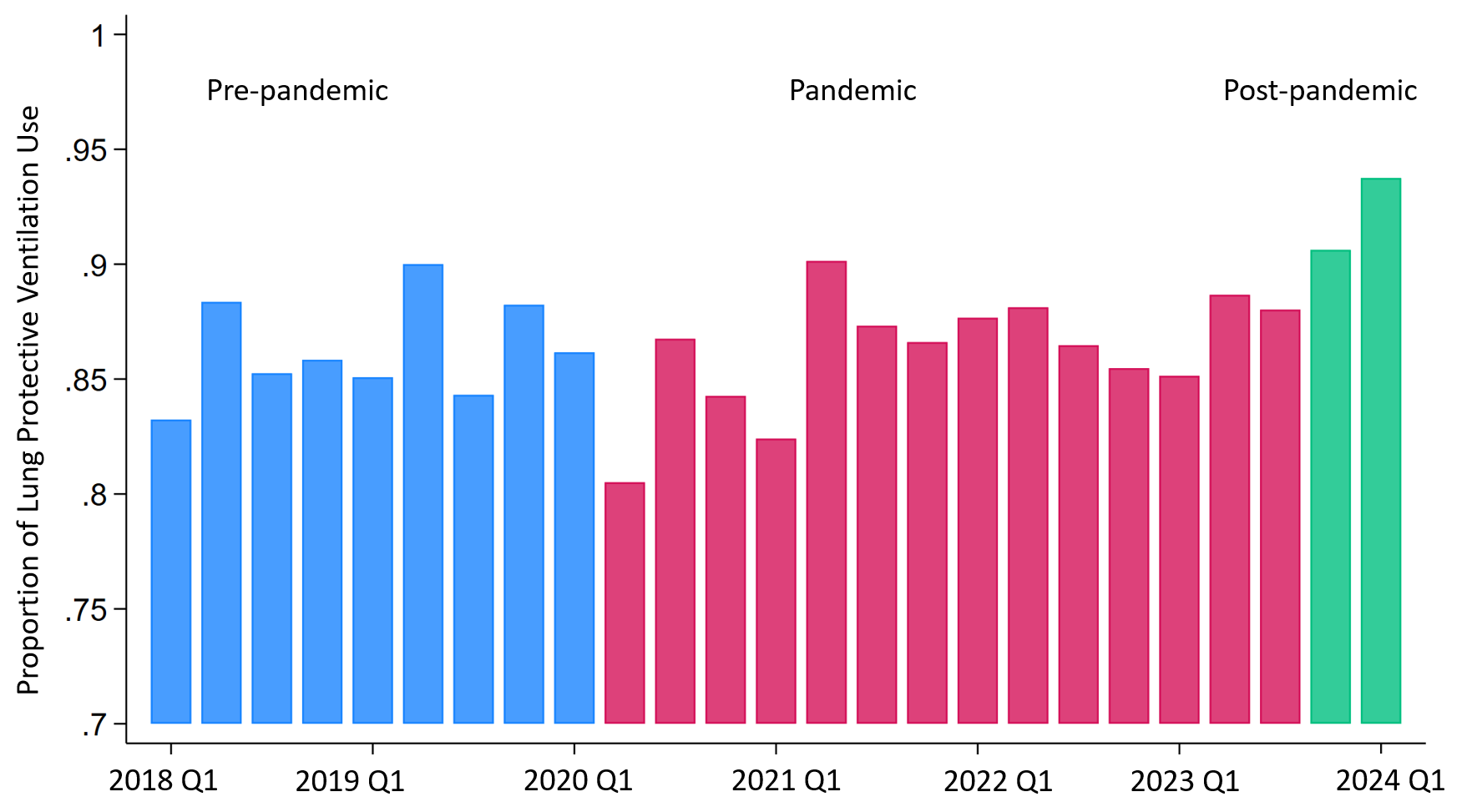
**

## **Figure S3. Interrupted Times Series Analyses Model**


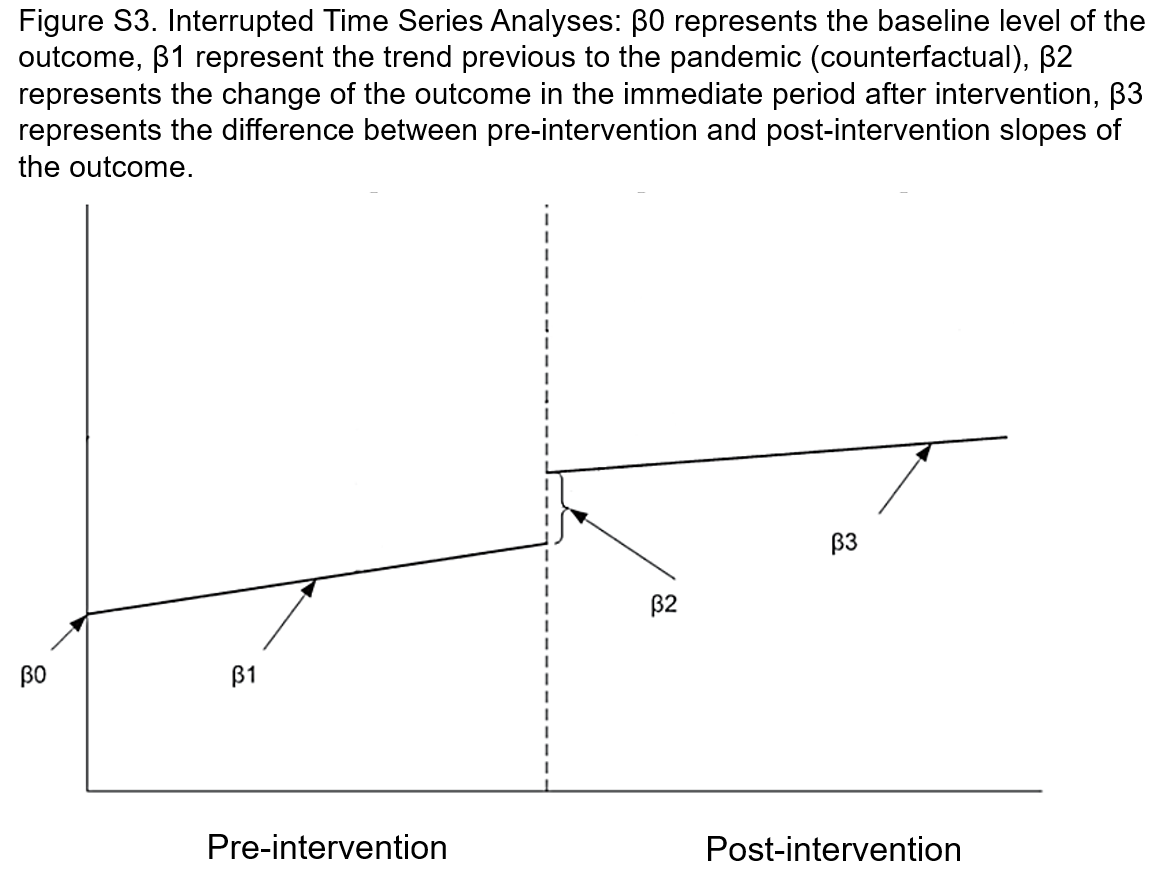


β0 represents the baseline level of the outcome, β1 represent the trend previous to the pandemic (counterfactual), β2 represents the change of the outcome in the immediate period after intervention, β3 represents the difference between pre-intervention and post-intervention slopes of the outcome.

**REFERENCES** 1. Azizi BA, Munoz-Acuna R, Suleiman A, Ahrens E, Redaelli S, Tartler TM, et al. Mechanical power and 30-day mortality in mechanically ventilated, critically ill patients with and without Coronavirus Disease-2019: a hospital registry study. J Intensive Care. 2023 Apr 6;11(1):14.

2. Munoz-Acuna R, Tartler TM, Azizi BA, Suleiman A, Ahrens E, Wachtendorf LJ, et al. Recovery and safety with prolonged high-frequency jet ventilation for catheter ablation of atrial fibrillation: A hospital registry study from a New England healthcare network. J Clin Anesth. 2024 May;93:111324.

3. Acute Respiratory Distress Syndrome Network, Brower RG, Matthay MA, Morris A, Schoenfeld D, Thompson BT, et al. Ventilation with lower tidal volumes as compared with traditional tidal volumes for acute lung injury and the acute respiratory distress syndrome. N Engl J Med. 2000 May 4;342(18):1301–8.

4. Elixhauser A, Steiner C, Harris DR, Coffey RM. Comorbidity measures for use with administrative data. Med Care. 1998 Jan;36(1):8–27.

5. Baum C, Schaffer M. ACTEST: Stata module to perform Cumby-Huizinga general test for autocorrelation in time series [Internet]. 2015 [cited 2024 Jan 24]. (Statistical Software Compo

6. Linden A. Conducting Interrupted Time-series Analysis for Single- and Multiple-group Comparisons. The Stata Journal. 2015 Jun;15(2):480–500.
7. Linden, A. (2017). A Comprehensive set of Postestimation Measures to Enrich Interrupted Time-series Analysis. The Stata Journal, 17(1), 73-88. doi:10.1177/1536867X1701700105nents). Available from: https://econpapers.repec.org/software/bocbocode/s457668.html
